# Theranostic ^64^Cu-DOTHA_2_-PSMA allows low toxicity radioligand therapy in mice prostate cancer model

**DOI:** 10.3389/fonc.2023.1073491

**Published:** 2023-01-18

**Authors:** Marie-Christine Milot, Ophélie Bélissant-Benesty, Véronique Dumulon-Perreault, Samia Ait-Mohand, Sameh Geha, Patrick O. Richard, Étienne Rousseau, Brigitte Guérin

**Affiliations:** ^1^ Department of Nuclear Medicine and Radiobiology, Faculty of Medicine and Health Sciences, Université de Sherbrooke, Sherbrooke, QC, Canada; ^2^ Sherbrooke Molecular Imaging Center (CIMS), Centre de recherche du CHUS, Sherbrooke, QC, Canada; ^3^ Department of Pathology, Faculty of Medicine and Health Sciences, Université de Sherbrooke, Sherbrooke, QC, Canada; ^4^ Department of Surgery, Division of urology, Faculty of Medicine and Health Sciences, Université de Sherbrooke, Sherbrooke, QC, Canada

**Keywords:** prostate cancer, radioligand therapy, copper-64, DOTHA_2_-PSMA, theranostic

## Abstract

**Introduction:**

We have previously shown that copper-64 (^64^Cu)-DOTHA_2_-PSMA can be used for positron emission tomography (PET) imaging of prostate cancer. Owing to the long-lasting, high tumoral uptake of ^64^Cu-DOTHA_2_-PSMA, the objective of the current study was to evaluate the therapeutic potential of ^64^Cu-DOTHA_2_-PSMA *in vivo*.

**Methods:**

LNCaP tumor-bearing NOD-*Rag1^null^IL2rg^null^* (NRG) mice were treated with an intraveinous single-dose of ^64^Cu-DOTHA_2_-PSMA at maximal tolerated injected activity, ^nat^Cu-DOTHA_2_-PSMA at equimolar amount (control) or lutetium-177 (^177^Lu)-PSMA-617 at 120 MBq to assess their impact on survival. Weight, well-being and tumor size were followed until mice reached 62 days post-injection or ethical limits. Toxicity was assessed through weight, red blood cells (RBCs) counts, pathology and dosimetry calculations.

**Results:**

Survival was longer with ^64^Cu-DOTHA_2_-PSMA than with ^nat^Cu-DOTHA_2_-PSMA (*p* < 0.001). Likewise, survival was also longer when compared to ^177^Lu-PSMA-617, although it did not reach statistical significance (*p* = 0.09). RBCs counts remained within normal range for the ^64^Cu-DOTHA_2_-PSMA group. ^64^Cu-DOTHA_2_-PSMA treated mice showed non-pathological fibrosis and no other signs of radiation injury. Human extrapolation of dosimetry yielded an effective dose of 3.14 × 10^-2^ mSv/MBq, with highest organs doses to gastrointestinal tract and liver.

**Discussion:**

Collectively, our data showed that ^64^Cu-DOTHA_2_-PSMA-directed radioligand therapy was effective for the treatment of LNCaP tumor-bearing NRG mice with acceptable toxicity and dosimetry. The main potential challenge is the hepatic and gastrointestinal irradiation.

## 1 Introduction

Prostate cancer is the second most common solid organ cancer in men worldwide and one of the most frequent causes of death by cancer in North America (1–3). There is still a lasting need for treatment options that will lead to better survival and lower side effects in men with metastastic disease (4–6). One promising avenue is radioligand therapy (4–6), which is the intravenous administration of a radioligand that binds to local and distant cancer metastases to irradiate them, usually with beta^-^ (β^-^) or alpha particles (7, 8). Affected organs and toxicity are dependent on the radioligand distribution (7, 8).

The prostate specific membrane antigen (PSMA) is an interesting target for radioligand therapy, given its important overexpression in most prostate cancer in comparison to normal tissue (8–10). Furthermore, PSMA small-size radioligands are internalized when bound, bringing them closer to the nucleus and thus, making DNA within reach of short-range decay particles like alpha particles and Auger electrons (7–9). Several radioligands have been proposed for PSMA radioligand therapy over the last decade, with small molecules showing the advantage of fast blood clearance over antibodies (9, 11). However, the modular nature of antibodies allowed the generation of smaller fragments, such as scFvs that can overcome some problems observed with antitbodies. Indeed, it has been shown that scFvs can offer appropriate tumor-to-background ratios for imaging and treating PSMA-expressing cancer (12).

The radioligand therapy current clinical arsenal is promising, even if there is still room for improvement. The small molecule PSMA-617 labeled with lutetium-177 (^177^Lu) (a low linear energy transfer (LET) β^-^ emitter) has been reported to decrease tumoral burden of metastatic, castration resistant prostate cancer with limited systemic side effects (13–15). It was approved in March 2022 for its treatment by the U.S. Food and Drug Administration (16) Its use is limited by ^177^Lu’s reactor production and there is also a significant proportion of resistance and relapse reported (5, 6, 11). The use of short range (50 to 100 µm), high LET, such as alpha particles emitter actinium-225 (^225^Ac) coupled to PSMA-617, was suggested for patients non-admissible or with refractory disease to radioligand therapy with a β^-^ emitter (5). Albeit studied in smaller cohorts, ^225^Ac-PSMA-617 proved efficacious (5, 17). Its use is limited given its association with long-lasting or severe xerostomia and limited current global availability of ^225^Ac (5, 17). Furthermore, actual therapeutic radioligands do not permit positron emission tomography (PET) imaging for patient selection, therapy planning, and follow-up. Hence, those radioligands are usually paired with similar diagnostic compounds such as gallium-68 (^68^Ga)-PSMA-617 (9, 10). However, the use of a different radiometal and sometimes different PSMA radioligands for theranostic applications may alter biodistribution and pharmacokinetics (18, 19).

**Figure 1:**
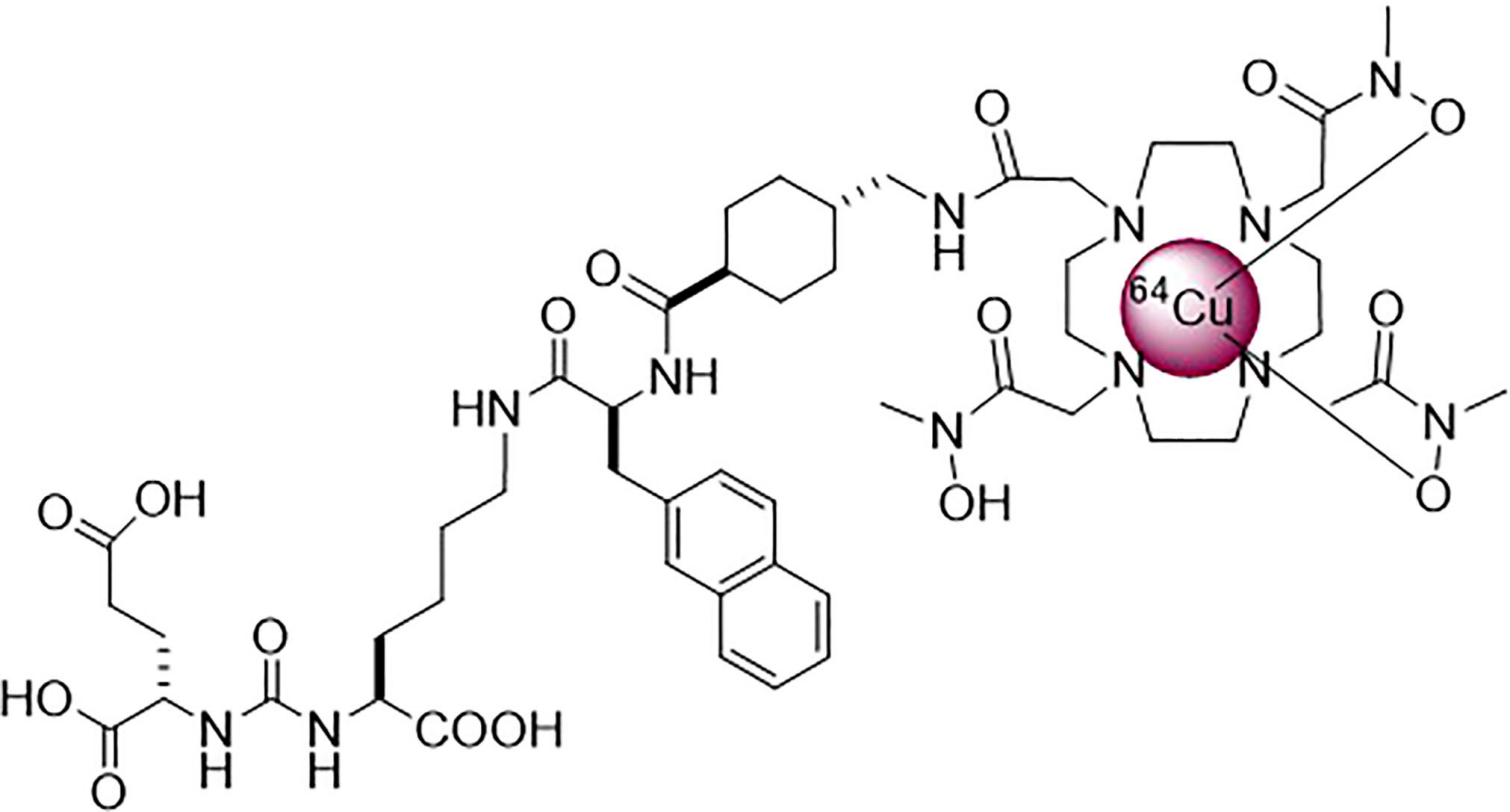
Structure of ^64^Cu-DOTHA_2_-PSMA.

In this project, we hypothesized that in addition to PET imaging, copper-64 (^64^Cu) could be of interest for radioligand therapy (20–22). ^64^Cu cyclotron production is easier than the production of ^67^Cu (20). The production route and mechanism of action (high LET Auger electron) of ^64^Cu feature a favourable supplement to therapeutic radionuclides such as ^177^Lu and ^225^Ac. Testing well-known (^177^Lu) and promising (^64^Cu) therapeutic radionuclides in a preclinical setting will allow us to select the most appropriate radioligand for optimal PSMA radioligand therapy. ^64^Cu has high LET, short range (~126 nm) Auger electron emissions (average energies (E_ave_) of 6.5 keV [22.7%] and 840 eV [57.4%]) and β^−^ emissions (39% [maximal energy (E_max_): 0.58 MeV, E_ave_: 0.19 MeV]) (20–22). These features offer therapeutic potential and possibly lower toxicity on non-target cells than exclusive or mainly β^−^ emitters (7, 22). ^64^Cu radioligand therapy was proven effective in preclinical context against various cancer types (23–30). For theranostic applications, ^64^Cu’s β^+^ enables PET imaging. (17.4% β^+^ [E_max_: 0.65 MeV, E_ave_: 0.28 MeV]). In this regard, we developed the radioligand ^64^Cu-DOTHA_2_-PSMA (**Figure 1**) to overcome ^64^Cu complexation challenges. Indeed, DOTHA_2_ offers fast complexation kinetics and high *in vivo* stability (31). It achieved high tumoral uptake up to 24h post-injection (p.i.) on preclinical PET imaging (31). ^64^Cu-DOTHA_2_-PSMA shown to be mainly cleared through the hepatobiliary pathway, which could be explained by its lipophilicity (log D = −0.96 ± 0.61) and its binding to plasma proteins; this was accompanied by a low kidney uptake and a fast-urinary clearance pattern (31). PSMA-617 radioligands have mainly urinary clearance with higher kidney uptake, especially at early time points (9).

The main goal of this study is to assess ^64^Cu-DOTHA_2_-PSMA’s therapeutic potential by determining its influence on survival and tumor size, as well as its general toxicity profile on organs of interest for PSMA radioligand therapy in comparison to control and to clinically used ^177^Lu-PSMA-617.

## 2 Materials and methods

### 2.1 Radioligand production

^64^Cu-DOTHA_2_-PSMA was synthesized, radiolabeled and characterized as previously described with a 116 ± 30 MBq/nmol molar activity (31).

^177^Lu-PSMA-617 preparation. The synthesis of the PSMA scaffold was achieved as previously described by Benešová et al. (32). Purity of the peptide was verified by HPLC and greater than 95% (**Supplementary Figures 1** and **2**). Both nuclear magnetic resonance spectroscopy and mass spectroscopy data were consistent with those reported in the literature (32). No-carrier added ^177^LuCl_3_ in 0.04 M HCl was obtained from Isotopia Molecular Imaging Ltd (Israël) and ITG Isotope Technologies Garching GmbH (Germany). Labeling of DOTA-PSMA was performed in 0.1 M ammonium acetate buffer pH 5.5 at 90°C for 15 min. Quality control of the drug was performed by Radio-TLC with sodium citrate buffer, pH 5.5, as eluent and always revealed labeling yields greater than 99% with an effective molar activity of 50 MBq/nmol. The radioligand solution was used without further purification steps.

### 2.2 Animal model

All experiments utilized NOD-*Rag1^null^* *IL2rg^null^* (NRG) mice (The Jackson Laboratory, 4-8 weeks old, and 18-26 g at time of tumor implantation) (33). For survival assays, human prostate adenocarcinoma LNCaP cells (ATCC) xenografts were implanted 4 ± 1 weeks before the experiment by injecting 200 µL of a 1:1 matrigel (Fisher Scientific)/phoshate-buffered saline (Wisent) mixture containing 8 × 10^6^ LNCaP cells subcutaneously on mice shoulders. LNCaP PSMA expression was previously verified (31). Protocols were approved by the Animal Ethics Committee of the Université de Sherbrooke according to the Canadian Council on Animal Care guidelines.

### 2.3 Maximal tolerated injected activity

To estimate maximal tolerated injected activity (IA) for survival assays, 6 NRG non-tumor-bearing mice were divided into 3 groups to test 70 MBq, 120 MBq and 150 MBq of ^64^Cu-DOTHA_2_-PSMA in 0.3 mL of saline (bolus injection by tail vein, constant molar activity). Starting IA was based on reported activities with ^64^Cu therapy in relatively similar conditions and scaled to mouse weight if needed (23–28). Mouse weight and general well-being were monitored daily 19 to 21 days. Maximal tolerated IA was chosen based on absence of general toxicity.

### 2.4 Survival experiments

When tumor reached a maximal diameter of 5 to 8 mm, LNCaP tumor-bearing mice (n = 30) were divided into 3 treatment groups with comparable tumor size distribution. NRG Mice were injected in the tail vein with 0.3 mL of saline containing previously determined maximal tolerated IA of ^64^Cu-DOTHA_2_-PSMA, equimolar amount of nonradioactive ^nat^Cu-DOTHA_2_-PSMA as a control or the maximum tolerated IA of ^177^Lu-PSMA-617 based on literature [120 MBq (34, 35)]. Survival end points were the ethical limit points: sustained weight loss > 20%, tumor diameter > 1 cm, behavior alterations suggestive of pain, tumor ulceration to the skin, non-manageable toxicity signs such as diarrhea or vomiting. Mouse weight, tumor size measured by caliper, and well-being were followed every 1 to 4 days for a maximum of 62 days. Tumor volume was calculated as an ellipsoid volume = (length × width × height)/2, estimating height = width (36). To determine the effect of radiotherapy on control of tumor growth, we suggest two calculated values: time-to-regrowth (TTR) for the interval to regrow after reaching the nadir and time-to-initial volume (TTIV) for the interval to regrow over the initial volume (calculations’ details in **Supplementary material**).

### 2.5 Toxicity assessment

General toxicity was assessed by the follow-up of the mouse weight. Mice food was mixed with water for treated mice. To obtain a general evaluation of blood toxicity, red blood cells (RBCs) were counted in survival experiment mice every 3 days from 2 to 18 days p.i. based on literature results (half the group per time point to spread out individual collections to every 6 days, leading in practice to 3 to 7 samples per time point) (sampling details in **Supplementary material**) (25, 26, 28). Results were compared to values measured in non-treated mice (n = 13).

To evaluate sub-acute toxicity, kidneys, liver and salivary glands of survival experiment mice were collected and stained with hematoxylin and eosin (H&E), Masson’s trichrome and light periodic acid-Schiff for the kidneys (preparation details in **Supplementary material**). Digitized slides were blindly analyzed by two persons (i.e. a certified pathologist and a medical student), to identify potential signs of radiation injury as suggested by Fajardo (37) and detailed in **Supplementary Table 1**. Changes could be parenchymal (e.g. necrosis, apoptosis, atrophy), stromal (e.g. fibrosis), vascular (e.g. edema, ischemia, hemorrhage, endothelial wall atypia and foam cells plaques) or inflammatory. Fibrosis was graded and averaged between results of both analysts if discordant. Proportion of live (at time of fixation) tumor cells over total tumor tissue was assessed using a grid. Results were compared between groups and to the results from two non-treated tumor-bearing mice ineligible to survival assays (oversize tumor).

### 2.6 Dosimetry

Kinetic values were obtained based on previously published healthy organ biodistribution and tumor PET data (31). Dosimetry was calculated for 25 g mouse model, human model, and sphere model for tumor in OLINDA/EXM 2.2.3 (Hermes Medical Solution). Detailed methodology is provided as supplementary material.

### 2.7 Statistical analysis

Results were reported as mean ± standard deviation unless mentioned otherwise. Tests used were two-tailed Student T test (Holm-Sidak correction for multiple comparisons, Welch test when applicable for non-consistent standard deviations) and two-tail correlation test with calculation of Pearson r coefficients. Survival results were analyzed by Kaplan-Meier curves stratified by treatment groups and comparison was made using Mantel-Cox log-rank test. For all tests, adjusted p < 0.05 was the threshold for significance. Excel and GraphPad Prism 8 were used for calculations.

## 3 Results

### 3.1 Maximal tolerated injected activity

**Figure 2:**
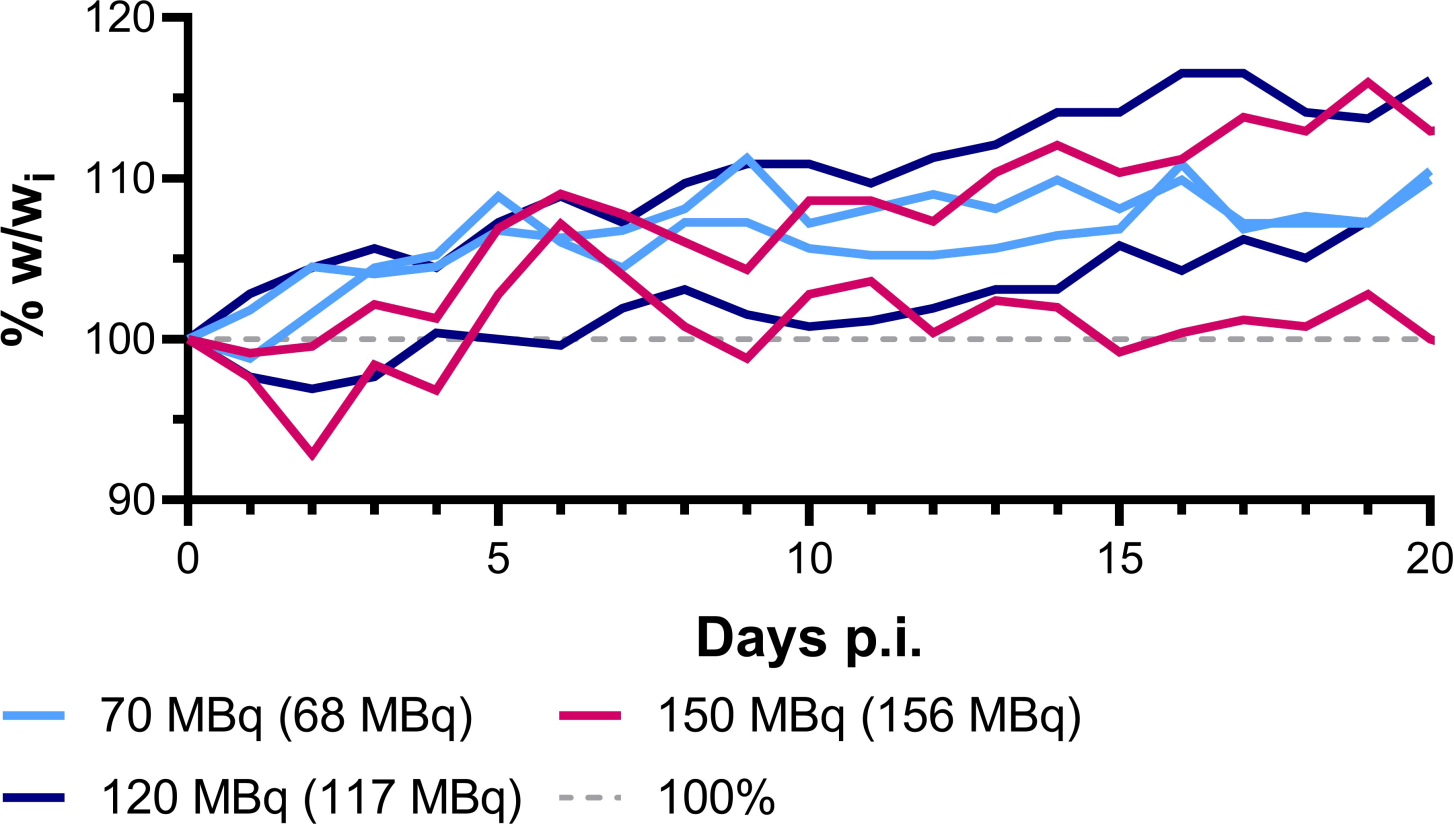
^64^Cu-DOTHA_2_-PSMA maximal tolerated IA experiment mice follow-up, weight (w) follow-up. (w_i_: initial weight).

In practice, IA tested were: 68.3 MBq (67.8 – 68.8 MBq, n = 2), 117 MBq (115 – 119 MBq, n = 2), and 156 MBq (154 – 159 MBq, n = 2) (**Figure 2** and **Supplementary Table 2**). Mice in the first two groups mainly gained weight from time of injection and showed normal behavior. One mouse in the last group lost up to 7% of its initial weight at first then stagnated around 100% of initial weight without external intervention. Mice from the last group showed signs of fear or stress for a total of 5 and 8 days out of 21 days follow-up but were active and vigorous. In consideration of these data and for practical reasons, we did not escalate to a further IA and determined 150 MBq as maximal tolerated IA to be injected in survival experiments.

### 3.2 Survival assays

**Figure 3:**
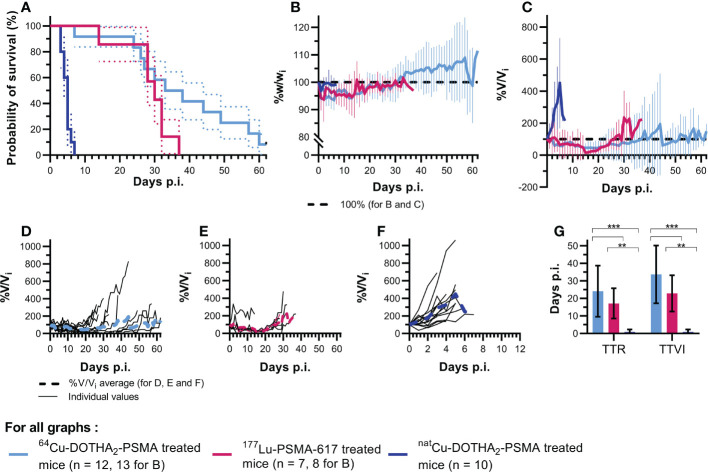
Survival assays results **(A)** Kaplan-Meier survival curves with significantly longer survival with ^64^Cu-DOTHA_2_-PSMA than with ^nat^Cu-DOTHA_2_-PSMA (p < 0.001, hazard ratio: 0.208 (95%CI: 0.0646 – 0.670) and no significant difference between ^64^Cu-DOTHA_2_-PSMA and ^177^Lu-PSMA-617 (p = 0.09, hazard ratio: 0.490 (95%CI: 0.165 – 1.45)), **(B)** Average weight evolution per group, **(C)** Average tumor size per group, **(D-F)** Individual tumor size for ^64^Cu-DOTHA_2_-PSMA, ^177^Lu-PSMA-617 and ^nat^Cu-DOTHA_2_-PSMA treated mice, respectively, **(G)** Time-to-regrowth (TTR) per group, **(H)** Time to initial volume (TTIV) per group. (V: tumoral volume, V_i_: initial tumoral volume. **p < 0.01, ***p < 0.001. Errors bars are standard errors for **(A)** and standard deviations for **B**, **C** and **G**).

Survival curves are displayed in **Figure 3A** and survival experiments details including mice and tumors individual characteristics are available in **Supplementary Tables 3**–**6**. Survival assays mice injected with 146 ± 14 MBq of ^64^Cu-DOTHA_2_-PSMA group (n = 12) survived significantly longer than controls injected with the same amount of ^nat^Cu-DOTHA_2_-PSMA (1.23 nmol, n = 10) (p < 0.001, hazard ratio: 0.208 (95%CI: 0.0646 – 0.670)). Their survival was not significantly different from mice injected with 123 ± 13 MBq of ^177^Lu-PSMA-617 (n = 7) (p = 0.09, hazard ratio 0.490 (95%CI: 0.165 – 1.45)). Median survival was 35.5 days with ^64^Cu- DOTHA_2_-PSMA (interquartile range (IQR): 26.5 days), 5 days with ^nat^Cu-DOTHA_2_-PSMA (IQR: 1 day) and 30 days with ^177^Lu-PSMA-617 (IQR: 4 days). Average tumor volumes at injection were 108 ± 55 mm^3^, 93.3 ± 56.1 mm^3^, and 107 ± 35 mm^3^ for ^64^Cu-DOTHA_2_-PSMA, ^nat^Cu-DOTHA_2_-PSMA and ^177^Lu-PSMA-617 groups, respectively (no significant difference between groups (p > 0.91)). One ^64^Cu-DOTHA_2_-PSMA treated mouse and one ^177^Lu-PSMA-617 treated mouse were retrospectively rejected from survival experiments after injection, as they should not have been included in the protocol because of an oversized tumor at the beginning of treatment. They were, however, included in toxicity results because this rejection criteria did not affect healthy organs or general well-being.

The cause of euthanasia was reaching tumor size limit for all but one mouse in ^177^Lu-PSMA-617 group (maximal weight loss, day 14) and two mice in the ^64^Cu-DOTHA_2_-PSMA group (pain behavior, day 60 and reaching follow-up limit, day 62). Two ^64^Cu-DOTHA_2_-PSMA and two ^nat^Cu-DOTHA_2_-PSMA mice had a second tumor of similar size at time of therapy that did not reach 10 mm tumor diameter limit, but mainly showed similar evolution patterns.

Individual and group average tumor volume progression is displayed in **Figures 3C–F**. In comparison to the control group, TTR and TTIV (**Figure 3G**) were significantly higher in groups treated with ^64^Cu-DOTHA_2_-PSMA (24.1 ± 14.6 days (p < 0.001) and 33.7 ± 16.5 days (p < 0.001), respectively) or with ^177^Lu-PSMA-617 (17.1 ± 8.6 days (p = 0.003) and 22.9 ± 10.4 days (p = 0.003), respectively). TTR and TTIV values for the control group were both of 0.857 ± 1.406 days. No statistically significant difference was observed between ^64^Cu-DOTHA_2_-PSMA and ^177^Lu-PSMA-617 (TTT: p = 0.26; TTIV: p = 0.24).

Within individual treatment groups ^177^Lu-PSMA-617 and ^64^Cu-DOTHA_2_-PSMA, there is no correlation between IA (absolute and per gram) and survival (R^2^ < 0.3). ^64^Cu-DOTHA_2_-PSMA treated tumors with bigger initial volume had shorter TTR, TTIV and survival, but correlation was low (R^2^ < 0.5). For ^177^Lu-PSMA-617 treated tumors, there were no correlation between initial tumor volume and survival or TTR, but there was a moderate positive correlation between initial tumor volume and TTIV (R^2^ = 0.611, p = 0.04). This correlation was mainly dependent on the mouse with the smallest initial tumor (49.0 mm^3^) being euthanized early for maximal weight loss and not for reaching the maximal tumor size. When testing without this mouse, there was no correlation (R^2^ = 0.179).

### 3.3 Toxicity assays in survival experiment mice.

General toxicity was assessed through weight follow-up. Only one mouse from the ^177^Lu-PSMA-617 group maintained a weight loss until reaching the ethical limit (20% weight loss). As depicted in **Figure 3B**, mice from all groups tended to lose weight at the beginning of study, but later maintained their weight or gained back at least their initial weight.

Normal range obtained from non-treated mice was 1.19 ± 0.21 × 10^10^ RBCs/mL (95% confidence interval (CI): 1.07 × 10^10^ to 1.30 × 10^10^ RBCs/mL, n = 13), **Supplementary Figure 3** and **Table 7**. Samples from ^64^Cu-DOTHA_2_-PSMA treated mice showed no significant difference from measured normal range, with values at days 8 and 17 lower than normal (all p > 0.35). Samples from ^177^Lu-PSMA-617 treated mice showed a significantly lower count on day 8 with 9.13 ± 0.49 × 10^9^ RBCs/mL (p < 0.001) and values staying low until day 14. Control ^nat^Cu-DOTHA_2_-PSMA treated mice RBCs counts could only be obtained on day 2 (n = 4) given the low median survival and did not significantly differ from normal range (p = 0.98).

**Figure 4:**
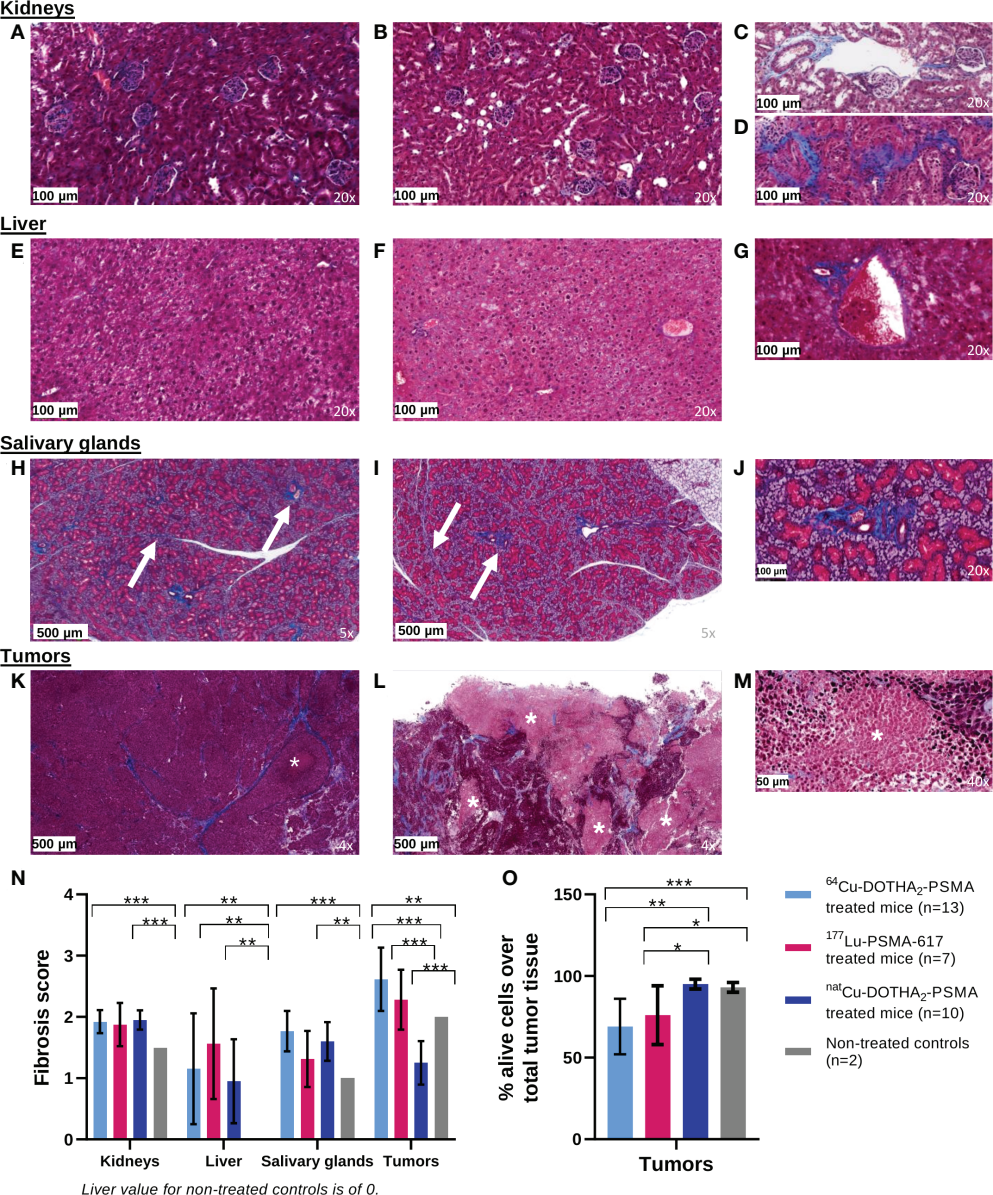
Pathology analysis results from controls, non-treated mice (left colum) in comparison to ^64^Cu-DOTHA_2_-PSMA treated mice (middle and right columns): Normal kidney histology was mainly found in controls **(A)** and treated mice **(B)**, with frequent non-pathological perivascular fibrosis **(C)** and rare, locally limited interstitial fibrosis **(D)**. Liver analysis also showed normal, non-injured tissue in controls **(E)** and treated mice **(F)** with some periportal fibrosis **(G)**. All salivary glands displayed non-pathological, expected fibrosis surrounding vessels and excretory duct and in septa in controls **(H)** and treated mice **(I, J)**. For LNCaP tumors, controls mainly showed alive tumor bulk, with fibrosis and infrequent necrosis **(K)**. In opposite, the irradiated tumors showed a lower proportion of alive cells and more necrosis **(L, M)**. Fibrosis scores by organs are presented in N and the proportion of alive tumor cells over total tumor tissue is presented in O. Stained by Masson’s trichome. Fibrosis: blue staining, arrow when needed. Necrosis in the tumor: lighter pink, asterix. In graphs, only significatively different relations are shown. *p < 0.05. **p< 0.01. ***p < 0.001.

Histopathological analysis of healthy organs showed little to no signs of radiotoxicity, as displayed in **Figure 4** for comparison between control, non-treated mice and ^64^Cu-DOTHA_2_-PSMA treated mice (detailed numerical values for all groups in **Supplementary Tables 8**, **9**). In kidneys (**Figures 4A–D**), liver (**Figures 4E–G**), salivary glands (**Figures 4H–J**), fibrosis increase in thickness and length was mainly found in the perivascular area, including the portal area for the liver and in addition to surrounding excretory ducts for the salivary glands (fibrosis score: **Figure 4N**). There were at most rare signs of interstitial fibrosis in the kidneys. In the liver, rare septal fibrosis was formed without important extensions between portal spaces and central veins to form nodules. Fibrosis was increased with ^64^Cu-DOTHA_2_-PSMA in comparison to the non-treated control in the kidneys, liver and salivary gland (p < 0.001, p = 0.006 and p < 0.001, respectively). For ^177^Lu-PSMA-617 group, fibrosis was significantly increased in comparison to non-treated controls in the liver (p = 0.009). For ^nat^Cu-DOTHA_2_-PSMA, fibrosis was increased in comparison to non-treated mice in the kidneys, liver and salivary glands (p < 0.001, p = 0.009 and p < 0.001, respectively). There were no significant differences between ^64^Cu-DOTHA_2_-PSMA, ^177^Lu-PSMA-617 and ^nat^Cu-DOTHA_2_-PSMA groups in all organs. In all healthy organs, there was neither necrosis, hemorrhage, cells or structure atrophy, edema (present surrounding the gland in one sample of ^nat^Cu-DOTHA_2_-PSMA), endothelial wall atypia or foam cells plaques, nor glomerular changes for the kidney. Limited, rare and locally circumscribed inflammation was noted in liver only, similar in all groups.

LNCaP tumors (**Figures 4K–M**) showed necrosis, apoptosis, fibrosis and bleeding, in higher proportion in tumors treated with radioactivity. Hence, the ratios of alive tumor cells to total tumor tissue in ^64^Cu-DOTHA_2_-PSMA and ^177^Lu-PSMA-617 groups were lower than with ^nat^Cu-DOTHA_2_-PSMA group (p < 0.001 and p = 0.04, respectively) and with non-treated control group (p = 0.002 and p = 0.04, respectively) (**Figure 4O**). Tumoral fibrosis was significantly increased in ^64^Cu-DOTHA_2_-PSMA and ^177^Lu-PSMA-617 groups in comparison to ^nat^Cu-DOTHA_2_-PSMA (p < 0.001 for both) and to non-treated mice for ^64^Cu-DOTHA_2_-PSMA (p = 0.005) (**Figure 4N**).

There was no correlation between IA or IA/g with fibrosis score per groups for all organs (R^2^: 0 – 0.38). In healthy organs, there was no correlation between survival (and therefore age) and fibrosis score (R^2^: 0.001 – 0.16). Concordance between the fibrosis score results of both analysts were of 85%, 82%, 67% and 67% for the kidneys, liver, salivary glands and tumors, respectively. Differences, when present, were of one point or less.

### 3.4 Dosimetry

Mouse dosimetry results are presented in **Table 1** with kinetics values used for calculations presented in **Supplementary Table 10**. Tumor dose was 2.67 × 10^2^ mGy/MBq (95% CI: 1.94 × 10^2^ – 3.4 × 10^2^ mGy/MBq), using average tumor weight of 0.1 g. In mouse, highest organ dose was estimated to the liver, leading to a tumor to liver ratio of 0.730 (0.464 – 1.09). Second highest ratio was to the stomach wall (estimated from stomach kinetics), at 1.84 (1.18 – 2.70). Tumor-to-kidney ratio was 2.23 (1.42 – 3.30).

**Table 1:** Mouse ^64^Cu-DOTHA_2_-PSMA dosimetry.

Target organs	Organ dose (mSv/MBq)	Organ dose minimal to maximal range (mSv/MBq)^a^
Liver	3.66E+02	(3.13E+02 – 4.18E+02)
Stomach Wall	1.45E+02	(1.26E+02 – 1.64E+02)
Large Int	1.30E+02	(1.14E+02 – 1.47E+02)
Small Intestine	1.30E+02	(1.14E+02 – 1.47E+02)
Kidneys	1.20E+02	(1.03E+02 – 1.37E+02)
Lungs	1.07E+02	(9.33E+01 – 1.20E+02)
Spleen	5.34E+01	(4.40E+01 – 6.29E+01)
Skeleton	5.05E+01	(3.43E+01 – 6.66E+01)
Thyroid	4.95E+01	(2.32E+01– 7.57E+01)
Pancreas	4.86E+01	(4.21E+01 – 5.50E+01)
Heart	4.34E+01	(3.29E+01 – 5.40E+01)
Testes	2.20E+01	(1.88E+01 – 2.53E+01)
Brain	1.27E+01	(1.05E+01 – 1.49E+01)
Total Body	5.11E+01	(4.32E+01 – 5.90E+01)
Tumor (0.1g) (mGy/MBq)	2.67E+02	(1.94E+02 – 3.40E+02)

a Minimal and maximal values were calculated from 95% confidence interval on biodistribution data.

Human extrapolation of dosimetry presented in **Table 2** (kinetic values in **Supplementary Table 11**) yielded effective dose of 3.14 × 10^-2^ mSv/MBq (95% CI: 2.67 × 10^-2^ – 3.61 × 10^-2^ mSv/MBq).

**Table 2:** Human dosimetry of ^64^Cu-DOTHA_2_-PSMA extrapolated from mouse biodistribution data.

Target Organs	Organ Dose (mSv/MBq)	Organ dose minimal to maximal range (mSv/MBq)^a^
Left colon	9.18E-03	(8.02E-03 – 1.03E-02)
Liver	6.37E-03	(5.46E-03 – 7.27E-03)
Lungs	5.37E-03	(4.82E-03 – 5.92E-03)
Stomach Wall	2.80E-03	(2.39E-03 – 3.21E-03)
Right colon	1.60E-03	(1.37E-03 – 1.83E-03)
Red Marrow	1.29E-03	(9.70E-04 – 1.60E-03)
Thyroid	8.52E-04	(4.07E-04 – 1.30E-03)
Rectum	5.98E-04	(5.09E-04 – 6.88E-04)
Kidneys	4.85E-04	(4.15E-04 – 5.55E-04)
Esophagus	4.65E-04	(3.84E-04 – 5.46E-04)
Testes	2.99E-04	(2.54E-04 – 3.45E-04)
Heart Wall	2.94E-04	(2.61E-04 – 3.27E-04)
Adrenals	2.91E-04	(2.02E-04 – 3.80E-04)
Gallbladder Wall	2.52E-04	(2.12E-04 – 2.93E-04)
Urinary Bladder Wall	2.35E-04	(1.79E-04 – 2.91E-04)
Spleen	2.16E-04	(1.78E-04 – 2.54E-04)
Pancreas	1.98E-04	(1.71E-04 – 2.24E-04)
Salivary Glands	1.88E-04	(1.62E-04 – 2.15E-04)
Small Intestine	1.73E-04	(1.45E-04 – 2.01E-04)
Osteogenic Cells	9.83E-05	(7.35E-05 – 1.23E-04)
Thymus	7.94E-05	(6.45E-05 – 9.43E-05)
Prostate	2.97E-05	(2.29E-05 – 3.65E-05)
Brain	2.96E-05	(2.52E-05 – 3.39E-05)
Effective Dose	3.14E-02	(2.67E-02 – 3.61E-02)

a Minimal and maximal values were calculated from 95% confidence interval on biodistribution data.

## 4 Discussion

The goal of this preclinical study was to evaluate ^64^Cu-DOTHA_2_-PSMA therapeutic potential based on its ability to increase survival and control tumor size with low toxicity profile and dosimetry. To our knowledge, this is the first study to evaluate ^64^Cu for PSMA radioligand therapy.

### 4.1 Maximal tolerated injected activity

^64^Cu-DOTHA_2_-PSMA maximal tolerated IA was estimated to be 150 MBq in NRG mice. This value is slightly higher than that reported for ^177^Lu-PSMA-617 in immunodeficient mice (34, 35) suggesting that the ^64^Cu-radioligand is well tolerated in immunodeficient mice.

### 4.2 Survival experiments

^64^Cu-DOTHA_2_-PSMA showed therapeutic efficacy. It showed survival and growth delay (TTR and TTIV values) superior to control and similar to ^177^Lu-PSMA-617, the lead radioligand for prostate cancer radioligand therapy. Other non-PSMA ^64^Cu radioligands in literature showed improvement in survival and tumor size control, but comparison is limited given their application with another target (23–30). For ^177^Lu-PSMA-617 with a slightly higher volume endpoint (1000 mm^3^), Fendler et al. found similar survival results to ours using 120 MBq (34) and Kuo et al. found higher median survival (58 days) even with the use of low IA (18.5 MBq) (38). Banerjee et al. found a 130 days median survival in a different prostate cancer model (PSMA+ PC3 PIP cells) with a higher tumoral volume endpoint (1800 mm^3^) (35).

### 4.3 Toxicity assessment

On the whole, toxicity results pointed to limited toxicity for ^64^Cu-DOTHA_2_-PSMA, similar to ^177^Lu-PSMA-617. Mice weight was maintained above limit endpoints for all but one ^177^Lu-PSMA-617 treated mouse, suggesting a low toxicity for both ^64^Cu-DOTHA_2_-PSMA and ^177^Lu-PSMA-617. Thought the only significant drop in red blood cells was observed in ^177^Lu-PSMA-617 mice samples at 8 days p.i., period of days 8 to 17 could be an interval of potential myelotoxicity for both radioactive compounds. Regarding kidneys, liver and salivary glands, only non-significant fibrosis in normal location was found with no other sign of radiation injuries nor pathological processes (e.g. nodule formation in liver, significant interstitial fibrosis in kidneys) (37, 39, 40). The difference between the control group injected with ^nat^Cu-DOTHA_2_-PSMA and the non-treated controls suggests an interaction of the ligand with the PSMA enzyme influencing its function (41). However, this is not likely to have important toxic implications at injected activities similar to the present study given that all fibrosis noted was non-pathological fibrosis. The fibrosis scale was obtained by blind comparison of samples, but on the whole the difference between scores were low and fibrosis was present in low quantity.

For LNCaP tumors, pathological analysis at varying time after implantation could have a confounder effect on the results. Non-treated LNCaP tumors can demonstrate necrosis and fibrosis without treatment (42). This phenomenon was witnessed in both control groups, but in a significantly smaller proportion than the ones noticed in the groups injected with radioactivity. In clinical pathology practice, important proportion of dead tumor cells is considered more likely to be induced at least in part by treatment. Furthermore, in mice treated with radioligands, necrosis was found over all tumor bulk and not only in the middle of a highly alive section. For healthy organs, few weeks difference in age between controls and treated mice is less likely to explain the difference between survival groups and non-treated controls since there is no correlation between time of death and fibrosis score.

For comparison regarding toxicity, preclinical studies of other ^64^Cu radioligand therapy compounds noted no to only transient weight loss and no to only mild signs in pathological examination (23–27, 29). Transient drop in RBCs was noted in a study 7 days p.i. at its highest IA (28) while no significant drop in RBCs was noted with others (23–26). For preclinical studies of ^177^Lu-PSMA-617, no weight loss was reported at a lower IA (38) and no change in RBCs was noted at 4 and 8 weeks p.i (34, 35).. With ^177^Lu-PSMA-617, Banerjee et al. noted mild changes in salivary glands, minimal changes in kidneys and significant changes in lacrimal glands and testes 8 weeks p.i (35).

### 4.4 Dosimetry

Dosimetry calculations yielded an effective dose within normal limits and showed that the main organs at risk for toxicity are the liver and gastrointestinal tract. High dose in liver for human and low tumor-to-liver ratio in mouse were expected from previous distribution results (31). Interestingly, kidneys and salivary glands dose estimations in humans were low for ^64^Cu-DOTHA_2_-PSMA, with favorable tumor-to-kidney ratio in mouse. In a theranostic perspective, human dosimetry estimation of ^64^Cu-DOTHA_2_-PSMA’s effective dose is within safe levels for imaging with diagnostic IA. For instance, if one was to inject 200 MBq as for ^68^Ga-PSMA-617 human scan, effective dose to the patient would be 6.28 mSv. This is similar to ^18^F-fluorodesoxyglucose PET for tumor imaging (~6.7 mSv), ^68^Ga-PSMA-11 and ^68^Ga-PSMA-617 (43–45). For comparison regarding therapy, human studies with ^177^Lu-PSMA-617 showed that organs receiving among the highest calculated doses are the kidneys (0.39 to 0.88 mGy/MBq) and salivary glands (0.44 to 1.17 mGy/MBq), respectively 7 to 16 times and 23 to 62 times higher values than the ^64^Cu-DOTHA_2_-PSMA human model absorbed doses estimations (46–48). Lacrymal gland dose estimation is unknown for ^64^Cu-DOTHA_2_-PSMA, since it is unavailable in the OLINDA/EXM human model used. It was, however, the highest dose in VISION phase III trial dosimetry sub-study (2.1 ± 0.5 mGy/MBq) (48). Liver, spleen and effective doses are similar for both radioligands, even with the important liver uptake for ^64^Cu-DOTHA_2_-PSMA (46, 47). Red marrow doses were higher for ^177^Lu-PSMA-617 than estimated for ^64^Cu-DOTHA_2_-PSMA, but this could be in part due to bone metastasis in patients for ^177^Lu-PSMA-617 (46–48).

Irradiation tolerance is considered to be higher for radioligand therapy than for external beam radiotherapy (47, 49). However, external beam radiotherapy dose limits are sometimes still used to guide radioligand therapy safety. Considering limits or standards of 23 Gy for the kidneys, 30 Gy for the liver, 20 − 25 Gy to the salivary glands, 2 Gy to the red marrow, 40 Gy to the intestines and 12 Gy for whole-lung irradiation (46, 47, 50–53), the injection of 44 GBq of ^64^Cu-DOTHA_2_-PSMA over several cycles such as for ^177^Lu-PSMA-617 (13) would not reach organ doses limits. Up to a total of 187 GBq could be injected before reaching the medullary and liver limit doses (calculations based on organ absorbed doses). We do not suggest using this high injected activity and it is simply underlined here to put ^64^Cu-DOTHA_2_-PSMA dosimetry profile in perspective. Clinical studies should be conducted to determine ^64^Cu-DOTHA_2_-PSMA biodistribution and dosimetry in humans to choose an appropriate injected activity.

Mouse dosimetry was obtained mainly to calculate ratios between tumor and healthy organs irradiation. In 2018, Kuo et al. reported mouse dosimetry for ^177^Lu-PSMA-617 with a methodology highly similar to ours using OLINDA/EXM 2.0 while we used the latest version 2.2.3 (38). Their results per organs for ^177^Lu-PSMA-617 were in average ~500 times lower than ours for ^64^Cu-DOTHA_2_-PSMA. We recalculated ^177^Lu-PSMA-617 dosimetry with OLINDA/EXM 2.2.3 based on their kinetics values and obtained results closer to ^64^Cu-DOTHA_2_-PSMA, notably 5.43 × 10^2^ mGy/MBq, 3.08 × 10^2^ mGy/MBq, and 5.85 mGy/MBq for tumor, kidneys and liver, respectively. Other organs doses ranged from 2.54 mGy/MBq to 14.2 mGy/MBq, except for the bladder dose calculated with a different voiding model than our methodology. The difference between ^177^Lu-PSMA-617’s results reported with OLINDA/EXM 2.0 and those obtained with the version 2.2.3 is notable. With 2.2.3, the tumor-to-kidney ratio is 1.76, slightly lower than for ^64^Cu-DOTHA_2_-PSMA, and the tumor-to-liver ratio is 92.8, importantly higher than for ^64^Cu-DOTHA_2_-PSMA.

Taking into consideration mouse and human models dosimetry results and analysis, the liver rather than the kidneys or the salivary glands would be at greater risk with ^64^Cu-DOTHA_2_-PSMA when compared to ^177^Lu-PSMA-617, though the liver dose is similar for both compounds. This dosimetry pattern could be a challenge for clinical use. On the other hand, it could also be of interest to complete the clinical arsenal with a radioligand with different toxicity profile to offer an endotherapeutic option to more patients (e.g. with renal insufficiency or important prior bladder irradiation) or for patients experiencing side effects (e.g. xerostomia). Their irradiation should also be considered as radioligand therapy is being increasingly studied at earlier stages of the disease (e.g. adjuvant to prostatectomy) (13). Patient living longer could experience different side effects over the long term or eventual use adjuvant to external beam radiotherapy could modify the risk profile, with increasing risk to pelvic organs in the irradiation field.

It is important to note that dosimetry estimates to organs and tumor do not take account of dose rates and repetitions of irradiation, which can both influence the therapeutic outcome and toxicity (23, 24, 28, 29, 54).

### 4.5 Limitations and variability

Some limitations and sources of variability should be underlined for the present study. Variation in group size can be explained by the few days variability in LNCaP xenograft growth parallel to radioisotope production and importation logistics. Variation in IA or tumor size at time of the experiment, which is expected for preclinical studies, did not significantly impact survival assays and toxicity results as verified by correlation tests. Fibrosis scores concordance rates between both analysts’ results were high to intermediate, with only one-point difference when present. Concordance in future studies results could be increased by making the fibrosis scale more precise thanks to the experience acquired in this project.

In hindsight, the implantation of one tumor per shoulder in the first treated mice was a suboptimal method. Although this is a common practice in LNCaP implantation to increase the probability of experiment success [estimated 50% success rate in xenografting (42, 55)], it also yielded more complicated survival analyses. For instance, if two tumors were present at the time of therapy, both did not necessarily reach the size limit on the very same day, even if response to treatment were mainly similar for both tumors. Consequently, four mice had to be euthanized when a first tumor reached the ethical limit, and therefore, growth follow-up for the second tumor was stopped and the time to size endpoint was not obtained. In an attempt to mitigate this issue, we selected TTIV and TTR methods for growth calculation, which also had the advantage to include in the analysis mice that reached limits other than tumor size. One tumor was implanted per mouse for subsequent radioligand therapy rounds.

The animal model has other limitations. The important immunosuppression required for tumor implantation in mice may lead to an underestimation of signs of toxicity through decreased inflammation (33). Furthermore, ^64^Cu-DOTHA_2_-PSMA binding can vary between mouse and human PSMA, which limits the interpretation of toxicity results in healthy PSMA-rich tissues. Although conduction of toxicity evaluation in survival assays mice is not ideal, it does allow for the assessment of a broad profile.

### 4.6 Perspectives

Future studies could evaluate the immunoreactivity of ^64^Cu-DOTHA_2_-PSMA as well as apoptosis and DNA double-strand breaks on tumor tissue after treatment. The PSMA expression and Ki67 marker expression could also be measured to confirm the level of PSMA positivity of surviving tumor cells and to have information on the proliferative capacity of the tumor cells respectively. Different injected activities and multidoses for radioligand therapy could be evaluated. Multidose of ^64^Cu-DOTHA_2_-PSMA could have an impact on tumor size control and toxicity, based on dose fractionation principles and similarly to previously published preclinical results with ^64^Cu and ^177^Lu and to ^177^Lu-PSMA-617 clinical protocols (14, 23–25, 27–30). Copper-67 (^67^Cu) has a similar β^-^ emission profile to ^177^Lu with a 2.6 days half-life and can be used in theranostic pair with copper-64 (^64^Cu) for PET imaging (20). ^64^Cu/^67^Cu-SAR-bisPSMA is currently under clinical trial for prostate cancer radioligand therapy (SECuRE trial, NCT04868604). Further research is also needed for a more complete toxicity profile, using healthy and immunocompetent mice followed over months with a wider panel of blood cells and specific time points of euthanasia for pathologic evaluation as groups. Other experiences could be added such as blood and urine markers of kidney and liver functions e.g. AST, ALT, blood urea nitrogen, creatinine and glomerular filtration rate. Finally, another interesting future research could evaluate the link between pathological findings, radioligand distribution in tissue, emission range and microdosimetry. For instance, the presence of radioligand in blood circulation could irradiate vasculature and be linked to perivascular fibrosis.

In conclusion, ^64^Cu-DOTHA_2_-PSMA showed efficiency for radionuclide therapy in comparison to control and similarly to the most clinically studied PSMA radioligand, ^177^Lu-PSMA-617. Insights on toxicity suggest a safety profile similar to the one of ^177^Lu-PSMA-617, but further confirmation studies are required. Dosimetry estimation yielded a low dose to the kidneys and salivary glands. Higher doses estimations were obtained for the liver and gastrointestinal tract, a perceived challenge to clinical use. Nevertheless, ^64^Cu-DOTHA_2_-PSMA offers the possibility to complement the current clinical arsenal with a theranostic radioligand that has different mechanism of action (both β^-^ particle and high LET Auger electron), clearance and dosimetry profile than previous radioligands. It could act as another alternative to ^177^Lu-PSMA-617, similarly to ^225^Ac-PSMA-617, to allow personalization of radioligand therapy based on patients’ comorbidities, prior treatments or experienced side effects.

## Data availability statement

The original contributions presented in the study are included in the article/**Supplementary Material**. Further inquiries can be directed to the corresponding author.

## Ethics statement

The animal study was reviewed and approved by Animal Ethics Committee of the Université de Sherbrooke according to the Canadian Council on Animal Care guidelines.

## Author contributions

M-CM: conceptualization, data acquisition, analysis, writing original draft, editing. OB-B and VD-P: conceptualization, data acquisition, review. SA-M: development, conceptualization, synthesis and chemical data acquisition, review. SG: pathology conceptualization and data analysis, review. PR: writing and review, supervision. ER: conceptualization, dosimetry data treatment and analysis, writing, review, supervision. BG: conceptualization, writing, review, editing, project management, supervision. All authors contributed to the article and approved the submitted version.
