# Supplementary material for: Theranostic 64Cu-DOTHA2-PSMA allows low toxicity radioligand therapy in mice prostate cancer model
Source: Front Oncol. 2023 Jan 18;13:1073491. doi: 10.3389/fonc.2023.1073491 (PMC9889868; doi:10.3389/fonc.2023.1073491)
Supplement: Supplementary file 1 [file DataSheet_1.pdf]

Supplementary Material

1 Supplementary protocol details for methodology

1.1 Definition details for time-to-regrowth (TTR) and time-to-initial volume (TTVI)

Time-to-regrowth (TTR) corresponded to the time needed for a tumor to reach a size at least 10% bigger than its nadir without going back below 10% of nadir. The 10% threshold was chosen to account for inherent small variations in tumor measurements. Average TTR was obtained per survival experiment group.

Time-to-initial-volume (TTIV) was the time for tumor size to rise higher than the initial volume. The last time point crossing the initial volume value was used (e.g. if a tumor size slightly increases just after injection before decreasing to a nadir, the last time point to cross over 100% without coming back below is used). Average TTIV was obtained per survival experiment group.

1.2 Red blood cells count sampling details

First, a red blood cells counting solution (Gower solution) was formed by the dilution of 0.09 g/ml of sodium sulfate in a 1:4 solution of glacial acetic acid (17.4 M) in water. Secondly, a maximum 10 µl was collected by a small cut under the mouse feet and a 1:4 dilution was obtained in heparin. Part of the collected blood mixed to heparin is diluted in the red blood cells counting solution to obtain a final dilution of 1:600 (4 µl of the blood/heparin solution in 596 µl of the red blood cells counting solution). Red blood cells were counted on a hemocytometer. Results were reported as number of RBCs per ml and compared to a normal range obtained by the same method in non-treated, same age, control NRG mice (n=13 samples).

1.3 Organs preparation for pathology analysis details

Kidneys, liver, salivary glands, and tumors were harvested immediately after euthanasia of survival experiment mice. Tissues were washed in 4°C PBS, placed in a histopathology box then submerged in 4°C 4% PFA for 16 hours. Thereafter, the samples were washed for 5 minutes twice, in a 70% ethanol bath, and were then stored at 4°C submerged in 70% ethanol until histologic cuts. Ethanol was changed weekly to ensure concentration remained appropriate. 4 µm histologic slices were obtained from paraffin blocks. Stains applied were hematoxylin and eosin (H&E), Masson’s trichrome and light periodic acid-Schiff for kidney only. Digitalized slides were blindly analyzed to identify the signs of potential radiation injury listed in supplementary Table S1 as suggested by Fajardo (1).

Supplementary table 1. Signs used in pathological analysis

Signs (scored as identified or absent unless specified otherwise)
Parenchymal changes
• Necrosis
• Apoptosis
• Atrophy, notably of renal tubules, hepatocytes or salivary glands
• For tumor only, proportion of cancer cells over total tumoral tissue
• Glomerular changes in kidneys
• Other
Stromal changes
• Fibrosis score and its localization
0) Absent
1) Mild
2) Moderate
3) Severe
4) Cirrhosis (for liver only)
Vascular changes and lesions (most commonly in capillaries and arterioles)
• Edema
• Ischemia
• Hemorrhage
• Thrombosis
• Endothelial and wall atypia
• Foam cells plaques
• Other
Inflammation, including immune cells infiltrate
Other

1.4 Dosimetry calculations

1) Data acquisition

For healthy organs, proportions of injected activity per gram (%IA/g) is obtained by biodistributions (results previously published, (2)). Data is corrected for physical decay to injection time. For tumor, %IA/cc is obtained from positron emission imaging.

2) Generation of time-activity curve and calculation of area under the curve

Physical decay is reapplied to %IA/g to represent time of death of the animal (1 h, 2 h, 4 h, 24 h or 48 h p.i.). A curve is plotted for each organ in Graphpad Prism 8 (X: time (h), Y: %IA/g. Area under the curve (AUC) is calculated by geometrical method from 0 h to 48 h or 0 to 24 h in tumor. To calculate the area under the curve from 48 h to infinity (24h to infinity for tumor), we assumed physical decay only using %IA/g at 48h (24h for tumor) as a starting point. Both values are added to form a total AUC, representing the total fraction of disintegrations per injected dose per gram of organ.

3) Mouse and tumor dosimetry calculation

Tumor kinetics was obtained by multiplying tumor AUC to the average tumor mass at time of radiotherapy and imaging (estimating from measured volume, assuming 1 g = 1 cm^3). In OLINDA/EXM 2.2.3, sphere model was used. Whole-body dosimetry for mouse was obtained by multiplying AUC by 25 g mouse model organs weight. Remainder of the body kinetic value was calculated from the muscle and biodistributed organs not included in OLINDA/EXM model. For muscle, lean mass weight of 19.2 g minus all known organs weight was used (9.46 g), therefore assuming muscle AUC to for organs with an unknown number of disintegrations.

4) Extrapolation to human

Using organ masses provided by OLINDA/EXM for the human male model IRCP 89, we extrapolated mice AUC to human proportions using Sparks method (1):

$$AUC_{human} = \frac{AUC_{mice} \times w_{human\ tissue} \times w_{total\ mice}}{w_{total\ human}} \quad (1)$$

Where “AUC” means “area under the curve”, “w” means “weight” for either a single human tissue (e.g., liver), mice total weight (25 g) or human total weight (73 kg). Skin, fat, muscle and seminal vesicles were not available in the OLINDA model. Average masses from the literature were used and their estimated human AUC were added to form “Remainder of the body” (or “Total body”), scaled for their human weight. Values used for model unknowns were: average fat mass of 19.4 kg, average muscle mass of 25.6 kg, average skin mass of 12.2 kg, and average seminal vesicles mass of 4.57 g.

5) Human dosimetry calculation

OLINDA/EXM 2.2.3 software (Hermes Medical Solution) was used to calculate doses. To calculate ^{64}Cu dose factor, OLINDA/EXM creator used emissions that contributed to 0.01% or more of the total decay scheme based on data from Brookhaven National Laboratory National Nuclear Data Center.

The average adult human male model IRCP 89 is used for healthy organ dosimetry. Blood AUC value was used for ”red marrow”. Stomach AUC value was used for “stomach contents”. Whole-heart AUC value was used for “heart wall” and blood AUC scaled to the heart-blood volume was used for “heart contents”. Bowel AUC value was an average value and is therefore scaled to the size of every section and used for the small intestine, the right colon, the left colon and the rectum. Bone AUC value was used for cortical and trabecular compartments and “value in bone volume” was selected. Gallbladder wall and urinary bladder wall values were unavailable due to difficulty with sampling. Other unavailable AUCs: eyes, prostate, thymus, esophagus.

6) Confidence intervals

95% confidence intervals (CI) was obtained on biodistribution data. Dosimetry was then calculated following the previously described method with the maximum and minimum from this interval for every organ.

2 Supplementary results

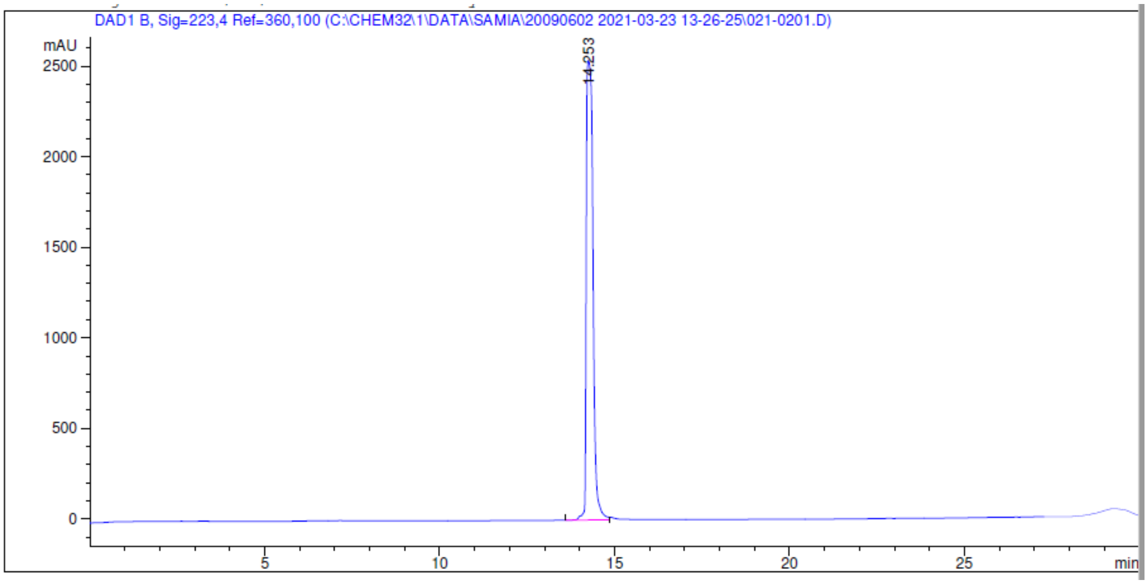

Supplementary figure 1. HPLC chromatogram for DOTA-PSMA

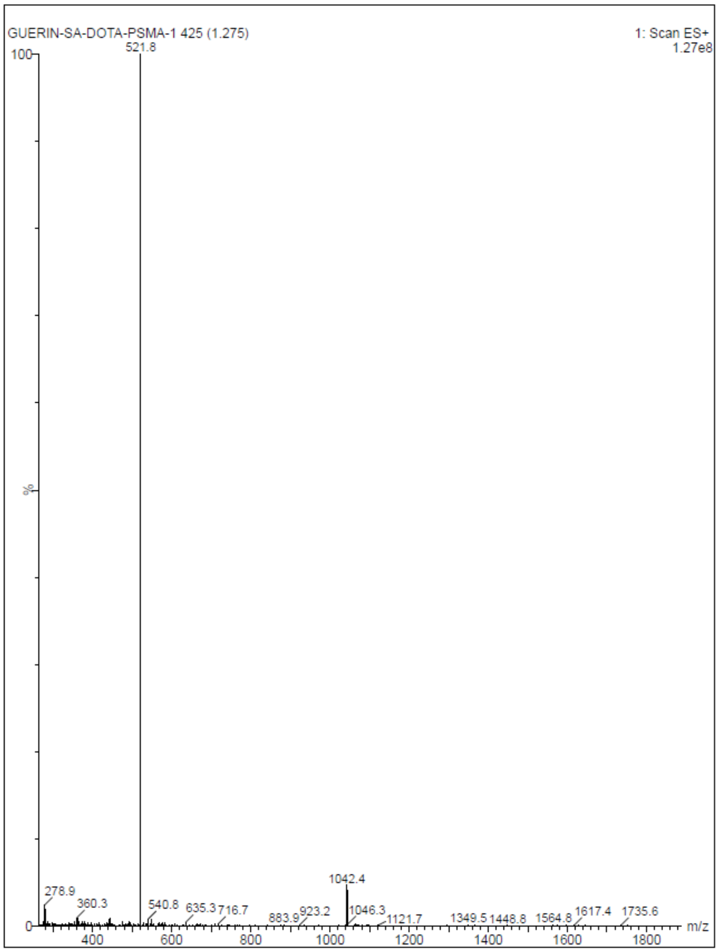

Supplementary figure 2. ESI-MS spectrum for DOTA-PSMA

Supplementary table 2. Weight follow-up for maximal tolerated injected activity assays mice

	70 MBq group		120 MBq group		150 MBq group	
Mice	1	2	3	4	5	6
IA (MBq)	67.8	68.8	119	115	154	159
Days p.i.	*Weights (g)*		*Weights (g)*		*Weights (g)*	
0	24.8	22.2	24.8	25.8	25.1	23.2
1	24.5	22.6	25.5	25.2	24.5	23.0
2	25.2	23.2	25.9	25.0	23.3	23.1
3	25.9	23.1	26.2	25.2	24.7	23.7
4	26.1	23.2	25.9	25.9	24.3	23.5
5	27.0	23.7	26.6	25.8	25.8	24.8
6	26.3	23.6	27.0	25.7	26.9	25.3
7	25.9	23.7	26.6	26.3	26.1*	25.0*
8	26.6	24.0	27.2	26.6	25.3	24.6
9	26.6	24.7	27.5	26.2	24.8*	24.2*
10	26.2	23.8	27.5	26.0	25.8	25.2*
11	26.1	24.0	27.2	26.1	26.0	25.2
12	26.1	24.2	27.6	26.3	25.2	24.9
13	26.2	24.0	27.8	26.6	25.7	25.6
14	26.4	24.4	28.3	26.6	25.6*	26.0*
15	26.5	24.0	28.3	27.3	24.9*	25.6
16	27.5	24.4	28.9	26.9	25.2	25.8*
17	26.5	23.8	28.9	27.4	25.4	26.4*
18	26.7	23.8	28.3	27.1	25.3*	26.2*
19	26.6	23.8	28.2	27.7	25.8	26.9*
20	27.4	24.4	28.8		25.1	26.2
21					24.8	26.3

* IA : injected activity, p.i. : post-injection.
† * : days when changed in behavior was noted.

Supplementary table 3. Survival experiments individual results for ^{nat}Cu-DOTHA$_2$-PSMA treated mice

Mice	Initial weight (g)	IA (MBq)	IA/g (MBq/g)	Survival (days p.i.)	Tumors identification	Initial volume (mm^3)	TTR (days p.i.)	TTIV (days p.i.)
1	25.2	N/A	N/A	5	1.1	66.6	0	0
2	24.6	N/A	N/A	5	2.1	132	0	0
3	17.5	N/A	N/A	5	3.1	104	3	3
4	19.5	N/A	N/A	5	4.1	23.9	0	0
					4.2	46.1	0	0
5	28.3	N/A	N/A	3	5.1	182	0	0
					5.2	17.8	0	0
6	23.5	N/A	N/A	6	6.1	100	0	0
					6.2	112	0	0
7	23.6	N/A	N/A	7	7.1	184	3	3
8	20.2	N/A	N/A	4	8.1	56.2	0	0
9	21.9	N/A	N/A	4	9.1	164	3	3
					9.2	76.9	3	3
10	22.2	N/A	N/A	3	10.1	40.4	0	0
Averages								
	22.7 ± 3.1	N/A	N/A	5 (median)<br>4.64 ± 1.22		93.3 ± 56.1	0.857 ± 1.406	0.857 ± 1.406

** IA : injected activity, p.i. : post-injection, TTR : time-to-regrowth, TTIV: time-to-initial volume.*

Supplementary table 4. Survival experiments individual results for ^{177}Lu-PSMA-617 treated mice

Mice	Initial weight (g)	IA (MBq)	IA/g (MBq/g)	Survival (days p.i.)	Tumor identification	Initial volume (mm^3)	TTR (days p.i.)	TTIV (days p.i.)
1	24.8	126	5.08	28	1.1	123	17	24
2	27.8	127	4.56	28	2.1	126	17	25
3	27.5	119	4.33	14	3.1	49.0	0	0
4	26.0	143	5.51	37	4.1	110	29	29
5	23.6	109	4.61	30	5.1	75.0	20	24
6	28.2	106	3.76	32	6.1	154	19	29
7	23.1	131	5.66	32	7.1	113	18	29
Averages								
	25.9 ± 2.1	123 ± 13	4.79 ± 0.67	30 (median)<br>28.7 ± 7.2		107 ± 35	17.1 ± 8.6	22.9 ± 10.4
				Rejected from survival assays				
8	22.6	139	6.13	N/A		322.9<br>(One diameter > 9 mm)	N/A	N/A

** IA : injected activity, p.i. : post-injection, TTR : time-to-regrowth, TTIV: time-to-initial volume.*

Supplementary table 5. Survival experiments individual results for ^{64}Cu-DOTHA$_2$-PSMA treated mice

Mice	Initial weight (g)	IA (MBq)	IA/g (MBq/g)	Survival (days p.i.)	Tumor identification	Initial volume (mm^3)	TTR (days p.i.)	TTIV (days p.i.)
1	22.2	145	6.53	24	1.1	203	7	16
2	23.0	134	5.84	7	2.1	213	6	6
3	21.4	147	6.89	27	3.1	69.2	7	
					3.2	147	16	24
4	20.0	149	7.47	62	4.1	34.1	57	59
					4.2	90.9	42	62
5	21.3	123	5.76	33	5.1	85.9	23	29
					5.2	143	17	31
6	22.0	147	6.69	57	6.1	98.4	31	47
7	23.0	156	6.78	38	7.1	104	22	35
8	27.5	158	5.73	60	8.1	29.0	38	51
9	24.3	143	5.89	26	9.1	121	13	22
10	21.9	141	6.45	49	10.1	140	37	42
11	23.5	130	5.54	30	11.1	91.3	20	20
12	25.3	175	6.92	44	12.1	45.5	26	28
Averages								
	22.9 ± 2.0	146 ± 14	6.38 ± 0.61	35.5 (median) 38.6 ± 16.5		108 ± 55	24.1 ± 14.6	33.7 ± 16.5
				Rejected from survival assays				
13	27.3	151	5.52	N/A	13.1	199 (One diameter > 9 mm)	N/A	N/A

** IA : injected activity, p.i. : post-injection, TTR : time-to-regrowth, TTIV: time-to-initial volume.*

Supplementary table 6. Survival assays average weights and tumoral volume evolution

	^{64}Cu-DOTHA$_2$-PSMA group						^{177}Lu-PSMA-617						^{nat}Cu-DOTHA$_2$-PSMA					
Days p.i.	Average tumor size (%V/Vi)			Average weight (%w/wi)			Average tumor size (%V/Vi)			Average weight (%w/wi)			Average tumor size (%V/Vi)			Average weight (%w/wi)		
0	100	±	0.0	100.0	±	0.0	100.0	±	0.0	100.0	±	0.0	100.0	±	0.0	100.0	±	0.0
1	88.7	±	33.9	97.2	±	3.6	83.0	±	43.4	94.2	±	4.5	139.2	±	58.6	96.9	±	3.7
2	94.0	±	30.4	97.3	±	3.4	107.1	±	101.7	93.7	±	8.1	176.2	±	121.0	99.2	±	2.4
3	59.4	±	27.5	94.2	±	3.0	108.7	±	92.1	98.0	±	3.1	289.6	±	169.1	99.7	±	2.2
4	68.2	±	24.9	94.8	±	3.5	90.8	±	69.7	97.4	±	3.0	326.2	±	226.4	99.6	±	4.9
5	58.1	±	30.2	94.0	±	3.8	64.2	±	79.6	96.1	±	2.8	449.0	±	281.7	98.9	±	3.7
6	56.6	±	37.3	93.5	±	3.7	68.9	±	91.2	96.8	±	3.3	249.8	±	38.2	98.9	±	2.7
7	78.2	±	47.1	96.4	±	3.0	68.8	±	89.3	95.1	±	3.1	214.4			99.2		
8	75.6	±	36.8	97.3	±	3.5	71.6	±	76.5	95.6	±	4.0						
9	81.5	±	30.5	96.4	±	3.5	63.1	±	57.0	95.2	±	4.2						
10	72.8	±	39.3	97.3	±	3.8	67.1	±	81.6	95.6	±	4.5						
11	55.0	±	20.8	96.2	±	3.6	60.1	±	52.6	94.6	±	4.7						
12	59.1	±	30.2	94.2	±	3.2	65.6	±	99.5	95.6	±	7.2						
13	52.0	±	30.4	94.2	±	3.2	55.3	±	87.9	94.5	±	6.7						
14	48.0	±	29.8	95.8	±	3.4	47.5	±	77.1	95.5	±	8.5						
15	45.7	±	27.8	95.7	±	3.5	16.1	±	13.8	100.8	±	6.8						
16	46.2	±	40.5	95.8	±	3.4	17.2	±	14.3	97.0	±	6.1						
17	47.2	±	39.2	96.4	±	3.5	21.9	±	19.9	98.9	±	2.5						
18	44.9	±	35.8	96.7	±	4.1	25.6	±	24.5	95.9	±	3.3						
19	44.3	±	45.8	98.2	±	5.6	26.1	±	22.0	97.6	±	4.1						
20	45.6	±	44.7	99.1	±	4.2	35.2	±	20.3	98.4	±	2.7						
21	52.2	±	58.1	98.3	±	4.5	47.8	±	32.2	98.1	±	2.1						
22	52.3	±	66.2	98.5	±	4.6	51.8	±	32.2	98.4	±	2.3						
23	58.3	±	75.0	98.4	±	4.7	57.8	±	34.6	99.9	±	3.9						

24	71.6	±	92.8	99.1	±	4.1	63.4	±	40.0	97.3	±	2.1
25	70.1	±	99.1	96.8	±	4.1	93.5	±	55.6	99.0	±	3.5
26	67.3	±	104.2	98.2	±	4.9	96.8	±	63.3	98.3	±	3.4
27	64.7	±	99.3	99.3	±	4.9	118.8	±	88.2	99.0	±	2.3
28	59.2	±	93.3	97.5	±	4.5	126.7	±	95.5	98.5	±	2.4
29	75.3	±	116.6	98.9	±	6.1	93.5	±	102.0	99.0	±	3.0
30	77.6	±	125.6	99.1	±	6.4	232.6	±	168.9	100.5	±	2.8
31	72.9	±	102.2	100.4	±	6.3	206.3	±	119.7	98.6	±	1.9
32	72.8	±	95.5	102.0	±	5.5	225.8	±	106.9	98.4	±	2.0
33	85.9	±	134.6	101.5	±	5.0	108.6			101.5		
34	82.0	±	172.1	103.7	±	5.9	153.0			99.6		
35	88.9	±	173.0	103.6	±	4.5	173.2			98.5		
36	93.1	±	171.0	103.1	±	4.2	217.0			97.7		
37	106.4	±	205.3	103.5	±	4.1	223.2			96.9		
38	149.8	±	214.4	104.2	±	6.5						
39	96.2	±	176.6	104.1	±	7.5						
40	109.7	±	209.8	104.7	±	7.3						
41	134.7	±	246.7	104.1	±	8.7						
42	143.1	±	255.2	105.3	±	9.5						
43	162.3	±	292.0	105.3	±	9.3						
44	191.3	±	317.5	103.6	±	9.0						
45	62.0	±	55.5	104.7	±	8.2						
46	66.1	±	63.0	104.9	±	8.3						
47	71.3	±	79.8	106.4	±	7.3						
48	78.7	±	87.6	105.8	±	7.1						
49	90.6	±	110.2	106.1	±	7.2						
50	80.8	±	91.9	104.3	±	10.8						
51	93.4	±	91.9	105.8	±	9.6						
52	129.8	±	126.0	104.7	±	11.4						

53	122.1 ± 120.2	106.7 ± 14.0		
54	119.0 ± 122.9	107.5 ± 14.2		
55	79.9 ± 80.2	107.3 ± 14.6		
56	135.8 ± 150.6	107.4 ± 13.4		
57	162.9 ± 132.0	108.8 ± 14.8		
58	100.3 ± 69.3	103.6 ± 15.4		
59	149.5 ± 100.8	100.4 ± 11.4		
60	105.5 ± 33.8	98.8 ± 13.7		
61	93.3 ± 37.3	108.5		
62	149.4 ± 45.9	111.5		

* If follow-up was exceptionally not done daily, measurements for the concerned mouse were assumed constant to calculate averages and draw graphs.

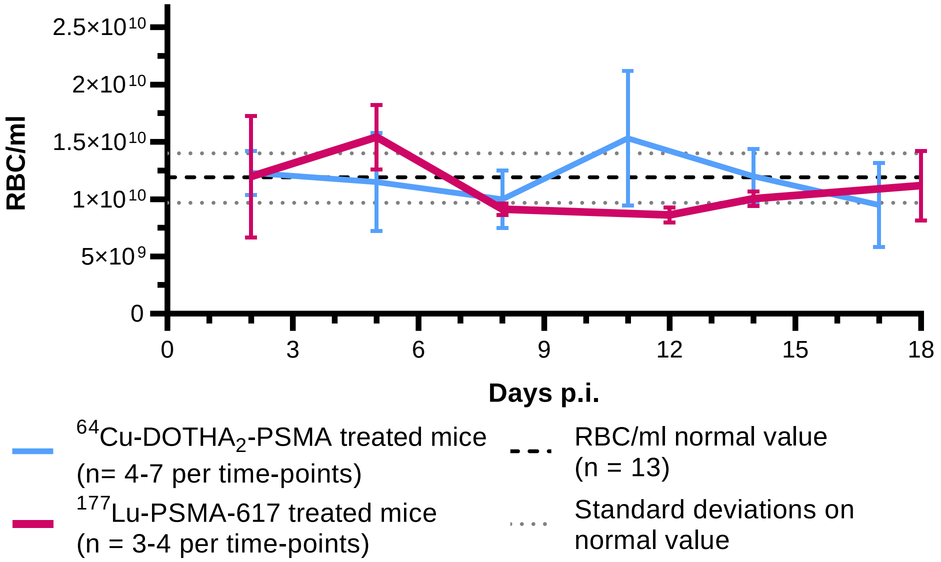

Supplementary figure 3. Red blood cells counts for mice injected with ^{64}Cu-DOTHA$_2$-PSMA and ^{177}Lu-PSMA-617

Supplementary table 7. Red blood cells counts in survival assays

^{64}Cu-DOTHA$_2$-PSMA group				^{177}Lu-PSMA-617 group			
Days p.i.	Average RBCs counts (RBCs/ml)		n	Days p.i.	Average RBCs counts (RBCs/ml)		n
2	1.23E+10 ±	1.91E+09	6	2	1.20E+10 ±	5.32E+09	4
5	1.15E+10 ±	4.28E+09	7	5	1.54E+10 ±	2.84E+09	4
8	1.00E+10 ±	2.52E+09	5	8	9.13E+09 ±	4.92E+08	4
11	1.53E+10 ±	5.88E+09	6	12	8.64E+09 ±	6.91E+08	4
14	1.20E+10 ±	2.40E+09	5	14	1.01E+10 ±	6.58E+08	3
17	9.48E+09 ±	3.68E+09	4	18	1.12E+10 ±	3.04E+09	4
^{nat}Cu-DOTHA2-PSMA group				Normal value			
Days p.i.	Average RBCs counts (RBCs/ml)		n	Days p.i.	Average RBCs counts (RBCs/ml)		n
2	1.19E+10 ±	2.31E+09	4	N/A	1.19E+10 ±	2.18E+09	13

Supplementary table 8. Numerical results from histopathology analysis of healthy organs

	Fibrosis score	Difference from nonradioactive control? (adjusted p)	Difference from non-treated controls? (adjusted p)	Other observations
Kidneys				
^{64}Cu-DOTHA$_2$-PSMA (n = 13)	1.95 ± 0.19	No	Yes, p < 0.001	
^{177}Lu-PSMA-617 (n = 8)	1.88 ± 0,35	No	No	No significant difference with ^{64}Cu-DOTHA$_2$-PSMA, p = 0,91
^{nat}Cu-DOTHA$_2$-PSMA, nonradioactive controls (n = 10)	1.95 ± 0.16	N/A	Yes, p < 0.001	
Non-treated controls (n = 2)	1.50 ± 0.00	Yes, p < 0.001	N/A	
	Other comments for kidneys : Fibrosis was mainly perivascular. No edema, necrosis, hemorrhage, inflammation or tubular atrophy were noted. Glomerular size was constant.			
Liver				
^{64}Cu-DOTHA$_2$-PSMA (n = 13)	1.15 ± 0.9	No	Yes, p = 0.006	
^{177}Lu-PSMA-617 (n = 8)	1.56 ± 0.9	No	Yes, p = 0.009	No significant difference with ^{64}Cu-DOTHA$_2$-PSMA, p = 0,56
^{nat}Cu-DOTHA$_2$-PSMA, nonradioactive controls (n = 10)	0.95 ± 0.69	N/A	Yes, p = 0.009	
Non-treated controls (n = 2)	0.00 ± 0.00	Yes, p = 0.009	N/A	
	Other comments for liver : Fibrosis was mainly perivascular, with some periportal fibrosis and rare septa. No necrosis, edema, hemorrhage or hepatocytes atrophy were noted.			
Salivary glands				
^{64}Cu-DOTHA$_2$-PSMA (n = 13)	1.77 ± 0.33	No	Yes, p < 0.001	
^{177}Lu-PSMA-617 (n = 8)	1.31 ± 0.46	No	No	No significant difference with ^{64}Cu-DOTHA$_2$-PSMA, p = 0,09
^{nat}Cu-DOTHA$_2$-PSMA, nonradioactive controls (n = 10)	1.60 ± 0.32	N/A	Yes, p = 0.001	Non-pathological edema in 1/10 specimen
Non-treated controls (n = 2)	1.00 ± 0.00	Yes, p = 0.001	N/A	
	Other comments for salivary glands: Fibrosis was mainly perivasuclar and around excretory ducts. No necrosis, no atrophy and no hemorrhage were noted.			

Supplementary table 9. Numerical results from histopathology analysis of LNCaP tumors

	Fibrosis score and proportion of alive tumor cells over whole tumor	Difference from nonradioactive control?	Difference from non-treated controls?	Other observations
^{64}Cu-DOTHA$_2$-PSMA (n = 13)	2,62 ± 0,52<br>69,2% ± 16,9 %	Yes, $p < 0.001$<br>Yes, $p < 0.001$	Yes, $p = 0.005$<br>Yes, $p = 0.001$	
^{177}Lu-PSMA-617 (n = 8)	2,28 ± 0,52<br>75,7% ± 18,1%	Yes, $p < 0.001$<br>Yes, $p = 0.04$	No<br>Yes, $p = 0.04$	No significant difference with ^{64}Cu-DOTHA$_2$-PSMA, p = 0,034 and 0,61
^{nat}Cu-DOTHA$_2$-PSMA, nonradioactive controls (n = 10)	1,25 ± 0,35<br>95,3% ± 3,1%	N/A<br>N/A	Yes, $p < 0.001$<br>No	
Non-treated controls (n = 2)	2,00 ± 0.00<br>93,1% ± 2,7%	Yes, $p < 0.001$<br>No	N/A<br>N/A	

Other comments for tumors: Tumor showed high grade characteristics, including frequent mitosis. Free red blood cells were noted, mainly in combination with necrosis. Necrosis was present and contributed to a decrease in alive tumor cells ratio.

Supplementary table 10. Mouse dosimetry kinetics for ^{64}Cu-DOTHA$_2$-PSMA

Source organs	Weights (g)	Kinetics Values (MBq-h/MBq)
Brain	0.46	3.3726E-02 (2.9705E-02 - 3.7752E-02)
LLI Contents	0.58	9.4085E-01 (8.2525E-01 - 1.0570E00)
Small Intestine	1.74	2.8091E00 (2.4640E00 - 3.1560E00)
Stomach Contents	0.055	7.6055E-02 (6.6976E-02 - 8.5079E-02)
Heart Contents	0.24	7.4283E-02 (5.0791E-02 - 9.7798E-02)
Kidneys	0.30	4.8316E-01 (4.1537E-01 - 5.5095E-01)
Liver	1.74	8.8687E00 (7.6073E00 - 1.0130E01)
Lungs	0.087	1.2794E-01 (1.1602E-01 - 1.3977E-01)
Pancreas	0.30	1.0703E-01 (9.5173E-02 - 1.1888E-01)
Cortical Bone	2.18	7.1420E-01 (4.5269E-01 - 9.7570E-01)
Spleen	0.11	7.3506E-02 (6.0411E-02 - 8.6613E-02)
Testes	0.16	3.6328E-02 (3.1801E-02 - 4.0872E-02)
Thyroid	0.014	9.9858E-03 (4.0227E-03 - 1.5943E-02)
Urinary Bladder Contents	0.060	N/A
Total	24.1	N/A
Tumor	0.1	3.9960E-01 (2.9008E-01 - 5.0920E-01)
Remainder of the body content		
Adrenals	0.012	5.9661E-03 (2.6184E-03 - 9.3125E-03)
Seminal Glands	0.16	1.8868E-02 (1.5041E-02 - 2.2694E-02)
Fat	4.80	5.2419E-01 (3.4146E-01 - 7.0658E-01)
Heart	0.12	9.1906E-02 (8.4424E-02 - 9.9387E-02)
Muscle	9.46 (lean mass weight minus all known organs)	1.3225E+00 (1.1263E+00 - 1.5176E+00)
Salivary Gland	0.15	9.6394E-02 (8.4007E-02 - 1.0880E-01)
Remainder of the body (sum of the above)		2.0598E00 (1.6539E00 - 2.4644E00)

* Values exceptionally presented with up to four decimals to facilitate reproduction.

Supplementary table 11. Human dosimetry kinetics for ^{64}Cu-DOTHA$_2$-PSMA

Source Organs	Weights (g)	Kinetics values (MBq-h/MBq)
Adrenals	14.0	2.3312E-03 (1.0231E-03 -3.6389E-03)
Brain	1450.0	3.5945E-02 (3.1659E-02 - 4.0236E-02)
Esophagus	40.0	0.0000E00 (0.0000E00 - 0.0000E00)
Eyes	15.0	0.0000E00 (0.0000E00 - 0.0000E00)
Gallbladder Contents	58.0	0.0000E00 (0.0000E00 - 0.0000E00)
Left colon	75.0	3.5897E-01 (3.1486E-01 - 4.0329E-01)
Small Intestine	350.0	8.2838E-02 (7.2660E-02 - 9.3068E-02)
Stomach Contents	250.0	7.0614E-02 (6.2184E-02 - 7.8993E-02)
Right colon	150.0	8.2838E-02 (7.2660E-02 - 9.3068E-02)
Rectum	75.0	3.8658E-02 (3.3908E-02 - 4.3432E-02)
Heart Contents	510.0	1.8400E-02 (1.2581E-02 - 2.4225E-02)
Heart Wall	330.0	9.0319E-02 (8.2966E-02 - 9.7671E-02)
Kidneys	310.0	1.7007E-01 (1.4621E-01 - 1.9394E-01)
Liver	1800.0	3.1477E00 (2.7000E00 - 3.5954E00)
Lungs	1200.0	6.0417E-01 (5.4788E-01 - 6.6006E-01)
Pancreas	140.0	1.6830E-02 (1.4966E-02 - 1.8694E-02)
Prostate	17.0	0.0000E00 (0.0000E00 - 0.0000E00)
Salivary Glands	85.0	1.9338E-02 (1.6853E-02 - 2.1826E-02)
Red Marrow	1170.0	1.2663E-01 (8.6586E-02 - 1.6672E-01)
Cortical Bone	4400.0	1.3464E-02 (8.5339E-03 - 1.8393E-02)
Trabecular Bone	1100.0	1.3464E-02 (8.5339E-03 - 1.8393E-02)
Spleen	150.0	3.3932E-02 (2.7887E-02 - 3.9983E-02)
Testes	35.0	2.7188E-03 (2.3799E-03 - 3.0589E-03)
Thymus	25.0	0.0000E00 (0.0000E00 - 0.0000E00)
Thyroid	20.0	4.8004E-03 (1.9338E-03 - 7.6642E-03)
Urinary Bladder Contents	211.0	N/A
Total body	73000.0	N/A
Remainder of the body		
Seminal glands	4.57	1.9004E-04 (1.5184E-04 – 2.2909E-04)
Fat	19440.0	7.2704E-01 (4.7360E-01 – 9.8001E-01)
Muscle	25550.0	1.2225E00 (1.0412E00 – 1.4029E00)
Skin	12160.0	9.0077E-01 (6.0869E-01 – 1.1924E00)
Remainder of the body (sum of the above)		2.8505E00 (2.1236E00 - 3.5756E00)

* Values exceptionally presented with up to four decimals to facilitate reproduction.

3 References

1. Fajardo LF. The pathology of ionizing radiation as defined by morphologic patterns. *Acta Oncol* (2005) 44:13–22. doi: 10.1080/02841860510007440

2. Milot M-C, Bélissant Benesty O, Dumulon-Perreault V, Ait-Mohand S, Richard PO, Rousseau É, Guérin B. 64Cu-DOTHA2-PSMA, a Novel PSMA PET Radiotracer for Prostate Cancer with a Long Imaging Time Window. *Pharmaceuticals* (2022) 15:996. doi: 10.3390/PH15080996
